# A Case of Neuralgia, Affecting the Branches of the Median Nerve after a Burn on the Thumb, and Which Was Cured under the Use of the Carbonate of Iron

**Published:** 1823-05

**Authors:** Henry Jeffreys

**Affiliations:** Surgeon of the St. George's and St. James's Dispensary, and Assistant Surgeon to the Lock Hospital.


**Art. III.**—*A Case of Neuralgia, affecting the Branches of the Median Nerve after a Burn on the Thumb, and which was cured under the Use of the Carbonate of Iron.* By Henry Jeffreys, Esq. Surgeon of the St. George's and St. James's Dispensary, and Assistant Surgeon to the Lock Hospital.

John Judd, thirteen years of age, in attempting to remove a saucer that had been standing for a length of time close to the fire, received a severe burn on the end of the right thumb. A vesication immediately arose on the injured part, to which some quack ointment was applied. The boy complained of much pain, and on the following day it was so much increased that his mother took him to an apothecary, who punctured the vesication, and directed a poultice to be applied. Under this application the vesication dried up; but the pain continued unabated, and in about a fortnight after the accident had occurred he was brought to my house, for my advice. At that time the pain had extended itself as high as the bend of the elbow, and evidently depended on injury of some nervous branch. It came on in paroxysms, which began in the burnt part, and proceeded along the ball of the thumb and the inner edge of the radius, in the course of the branches of the median nerve, up to the elbow. The pain was compared to the running of needles, or some sharp instrument, through the part. The attacks came on at irregular intervals, but were most frequent, and of longest duration, in the evening; and they frequently awoke him out of his sleep in the night. During a paroxysm, the thumb was often affected with involuntary twitchings and agitation. The slightest pressure on the burnt part brought on an attack of pain; and the same effect was produced by pinching up, or even touching, the skin in the course of the disordered nerves. There was a strongly-marked expression of suffering depicted in the boy's countenance, but his general health was apparently unaffected. The end of the thumb was still covered with a pellicle of thick, dry, brown-coloured cuticle: this was removed, and the part beneath, as well as the rest of the thumb, was, to all appearance, in a perfectly healthy condition.

The thumb was wrapped up in soap-plaster; and he was directed to take Dover's powder, and the extracts of hemlock and henbane. For a day or two after he began this plan, the paroxysms of pain were less frequent, and more bearable; but they soon relapsed into their former severity, and he was therefore put upon the carbonate of iron, of which he took a scruple three times a-day.

At the end of a week, the attacks had become much milder, and extended no further than the wrist. The pain, however, could still be excited by pressing or pinching the skin, even as high as the elbow. The dose of the medicine was increased to half a drachm, and agreed very well. At the expiration of a fortnight, the paroxysms had ceased, and he suffered no pain except when pressure was made upon the skin in the course of the injured nerves. This effect also soon subsided, and at the end of six weeks, being quite well, the medicine was left off.

I desired him to call on me without delay, if the pain should threaten to return; but three months have elapsed since I discontinued my attendance, and I have heard nothing from him.

*Clarges-street; April* 7, 1823.

